# Dopamine Oxidation and Autophagy

**DOI:** 10.1155/2012/920953

**Published:** 2012-08-26

**Authors:** Patricia Muñoz, Sandro Huenchuguala, Irmgard Paris, Juan Segura-Aguilar

**Affiliations:** ^1^Molecular & Clinical Pharmacology, ICBM, Faculty of Medicine, University of Chile, Independencia 1027, Santiago 8380453, Chile; ^2^Department of Basic Sciences, Santo Tomas University, Viña del Mar 2561780, Chile

## Abstract

The molecular mechanisms involved in the neurodegenerative process of Parkinson's disease remain unclear. Currently, there is a general agreement that mitochondrial dysfunction, **α**-synuclein aggregation, oxidative stress, neuroinflammation, and impaired protein degradation are involved in the neurodegeneration of dopaminergic neurons containing neuromelanin in Parkinson's disease. Aminochrome has been proposed to play an essential role in the degeneration of dopaminergic neurons containing neuromelanin by inducing mitochondrial dysfunction, oxidative stress, the formation of neurotoxic **α**-synuclein protofibrils, and impaired protein degradation. Here, we discuss the relationship between the oxidation of dopamine to aminochrome, the precursor of neuromelanin, autophagy dysfunction in dopaminergic neurons containing neuromelanin, and the role of dopamine oxidation to aminochrome in autophagy dysfunction in dopaminergic neurons. Aminochrome induces the following: (i) the formation of **α**-synuclein protofibrils that inactivate chaperone-mediated autophagy; (ii) the formation of adducts with **α**- and **β**-tubulin, which induce the aggregation of the microtubules required for the fusion of autophagy vacuoles and lysosomes.

## 1. Dopamine Synthesis and Degradation

**Figure 1:**
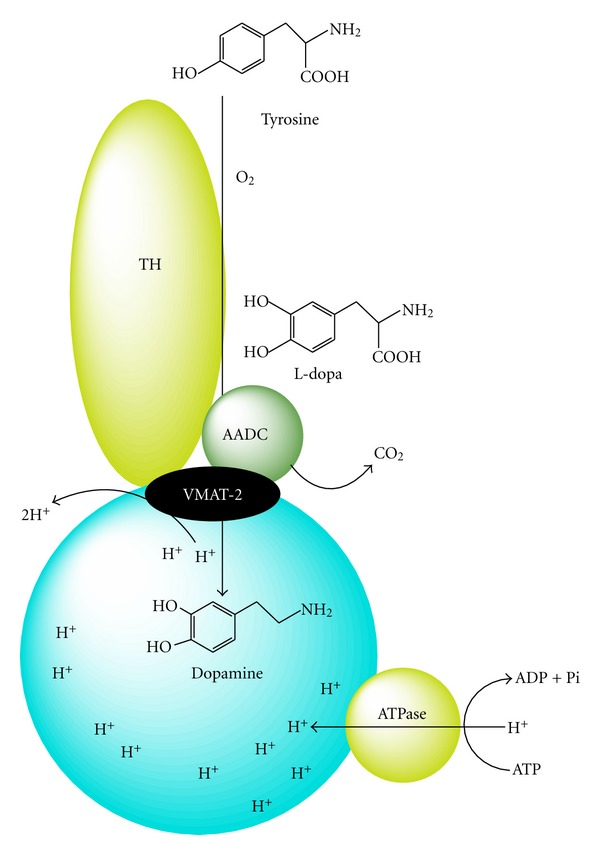
Dopamine synthesis. Synthesis of dopamine catalyzed by tyrosine hydroxylase (TH) and aromatic amino acid decarboxylase (AADC), which are both associated with the vesicular monoaminergic transporter-2 (VMAT-2).

Dopamine is a neurotransmitter that plays an essential role in the control of movements and loss of dopaminergic neurons containing neuromelanin in the nigrostriatal system. In addition, dopamine is involved in the development of motor symptoms experienced in patients diagnosed with Parkinson's disease (PD). Dopamine is synthesized in a sequential reaction in which the cytosolic enzymes tyrosine hydroxylase (TH) and aromatic amino acid decarboxylase (AADC) catalyze the hydroxylation of the amino acid tyrosine to L-dihydroxyphenylanaline (L-dopa) and decarboxylation of L-dopa to dopamine, respectively. The protons of the hydroxyl group in dopamine dissociate when dopamine is localized in the cytosol at physiological pH. However, these protons are tightly bound to the hydroxyl group once dopamine is inside monoaminergic synaptic vesicles, which have a relatively low pH. The membrane of monoaminergic synaptic vesicles contains a vesicular monoaminergic transporter-2 (VMAT-2) that catalyzes the uptake of dopamine into these vesicles. These monoaminergic synaptic vesicles contain an ATPase that hydrolyzes ATP to ADP and Pi, and one proton (H^+^) is translocated into the vesicle, generating a proton gradient. VMAT-2 uses this proton gradient to take up one molecule of dopamine with the concomitant release of two protons [1, 2]. The increase of protons inside monoaminergic synaptic vesicles induces a decrease in the pH of the vesicle, which is estimated to be 2 to 2.4 pH units lower than that of the cytosol [3]. TH and AADC have been shown to associate with monoaminergic synaptic vesicles containing VMAT-2 [4] by forming a complex. Tyrosine is then converted to L-dopa and immediately decarboxylated to dopamine, preventing the presence of free dopamine in the cytosol (Figure 1).

**Figure 2:**
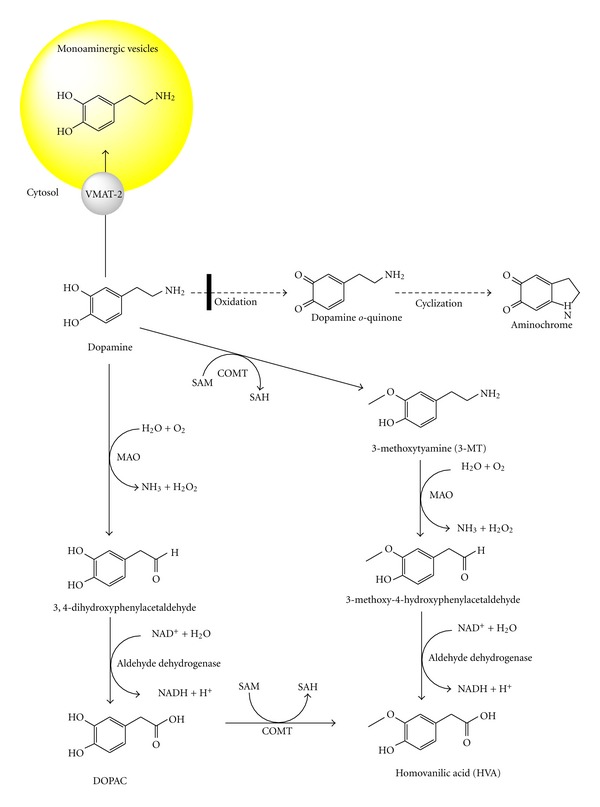
Dopamine degradation catalyzed by MAO and COMT. Dopamine oxidation to aminochrome is prevented by dopamine degradation mediated by both MAO and COMT. MAO catalyzes the oxidative deamination of dopamine amino group to 3,4-dihydroxyphenylacetaldehyde, that is converted to 3,4-dihydroxyphenylacetic acid (DOPAC) catalyzed by aldehyde dehydrogenase. COMT catalyzes the methylation of dopamine to 3-methoxytyramine that is substrate for MAO that catalyzes the formation of 3-metoxy-4-hydroxyphenylacetaldehyde. Homovanillic acid is formed when MAO uses 3-metoxy-4-hydroxyphenylacetaldehyde as substrate or when DOPAC is metabolized by COMT.

Dopamine in the cytosol spontaneously oxidizes to aminochrome without metal-ion catalysis [5]. Thus, VMAT-2 plays an important role in preventing the oxidation of dopamine in dopaminergic neurons. Other enzymes that prevent dopamine oxidation to aminochrome are monoamino oxidase (MAO) and catechol ortho-methyl transferase (COMT). MAO degrades excess dopamine in the cytosol by catalyzing the oxidative deamination of the amino group of dopamine to 3,4-dihydroxyphenylacetaldehyde with the concomitant formation of an ammonium molecule and hydrogen peroxide. Aldehyde dehydrogenase can then convert 3,4-dihydroxyphenylacetaldehyde to 3,4-dihydroxyphenylacetic acid (DOPAC), which can be converted to homovanillic acid catalyzed by COMT. Dopamine can also be methylated by COMT, generating 3-methoxytyramine, which can be converted to 3-methoxy-4-hydroxyphenylacetaldehyde, hydrogen peroxide, and NH_3_ by the enzyme MAO. Finally, the enzyme aldehyde dehydrogenase catalyzes the conversion of 3-methoxy-4-hydroxyphenylacetaldehyde to homovanillic acid (Figure 2). MAO enzymes are localized in the outer membranes of mitochondria in neurons, glial cells, and other cell types [6, 7]. MAO-A is mostly localized in catecholaminergic neurons, whereas MAO-B is found in serotonergic and histaminergic neurons as well as astrocytes [8]. COMT has two isoforms, one soluble (S-COMT) and one membrane-bound (MB-COMT) isoform. Both isoforms are found in microglial, astroglial, and some neuronal cells, such as pyramidal neurons, cerebellar Purkinje and granular cells, and striatal spiny neurons [9]. However, dopamine still oxidizes to aminochrome, even in the presence of VMAT-2, MAO-A, and S-COMT, which prevent the existence of free dopamine in the cytosol. Aminochrome, the precursor to neuromelanin, is a dark pigment found in dopaminergic neurons localized in the substantia nigra.

## 2. Dopamine Oxidation

**Figure 3:**
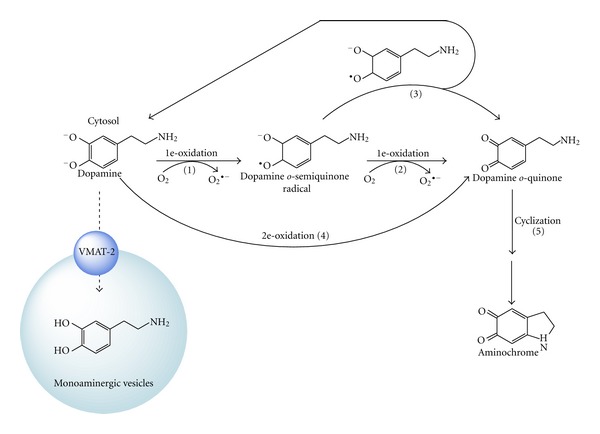
Dopamine oxidation to aminochrome at physiological pH. Dissociated dopamine can be oxidized to a dopamine *o*-semiquinone radical by the reduction of one molecule of oxygen to form a superoxide radical. The dopamine *o*-semiquinone radical can then disproportionate with another dopamine *o*-semiquinone radical, generating one molecule of dopamine and one molecule of dopamine *o*-quinone. Alternatively, the dopamine o-semiquinone radical can undergo one-electron oxidation to dopamine *o*-quinone by reducing one molecule of molecular oxygen to a superoxide radical. Dopamine *o*-quinone immediately cyclizes to form aminochrome, which is only stable in environments below pH 2.

Free cytosolic dopamine has protons that dissociate from their corresponding hydroxyl groups, promoting the oxidation of dopamine to dopamine *o*-quinone. This oxidation can proceed via a one-electron oxidation of dopamine to form a dopamine *o*-semiquinone radical (reaction 1), which is subsequently oxidized to dopamine *o*-quinone (reaction 2) by reducing two molecules of oxygen to superoxide radicals. The dopamine o-semiquinone radical does not strongly react with oxygen, leading to the formation of leukoaminochrome o-semiquinone radical during a one-electron reduction of aminochrome [10]. Subsequently, two dopamine *o*-semiquinone radicals can disproportionate, generating one molecule of dopamine *o*-quinone and one molecule of dopamine (reaction 3). A two-electron oxidation of dopamine to dopamine *o*-quinone is catalyzed by the enzyme tyrosinase. Notably, the presence of dopamine *o*-semiquinone is not detected by electron spin resonance [11]. Dopamine *o*-quinone is not stable in the cytosol at physiological pH and its amino chain cyclizes (reaction 5), generating aminochrome (Figure 3). Dopamine *o*-quinone has been reported to form adducts with parkin, mitochondrial complex I and III, and dopamine transporters [12–14]. However, the molecule actually that forms these adducts is aminochrome because dopamine *o*-quinone is only stable below pH 2.0 [11]. Aminochrome is formed by the oxidation of dopamine by tyrosinase, which can be further purified by chromatography, and it is stable for approximately 3 hours [15].

The oxidation of dopamine can also be catalyzed by enzymes with peroxidase activity, such as prostaglandin H synthase, cytochrome P450 forms, dopamine **β**-mono-oxygenase, and xanthine oxidase [16–20]. Lactoperoxidase catalyzes the one-electron oxidation of dopamine to a dopamine *o*-semiquinone radical, which was confirmed by electron spin resonance [10]. However, dopamine can also be oxidized by metals, such as manganese, copper, iron, or sodium metaperiodate [11, 21–24]. At physiological pH, dopamine o-quinone is a transient product because it is unstable above pH 2, resulting in the further oxidation of dopamine [11]. Dopamine *o*-quinone rearranges by cyclizing its amino chain to form aminochrome (reactions 5 and 6). These proteins are inactivated by aminochrome because dopamine *o*-quinone cyclizes immediately at physiological pH [11].

## 3. Aminochrome Metabolism

### 3.1. Formation of Neuromelanin

**Figure 4:**
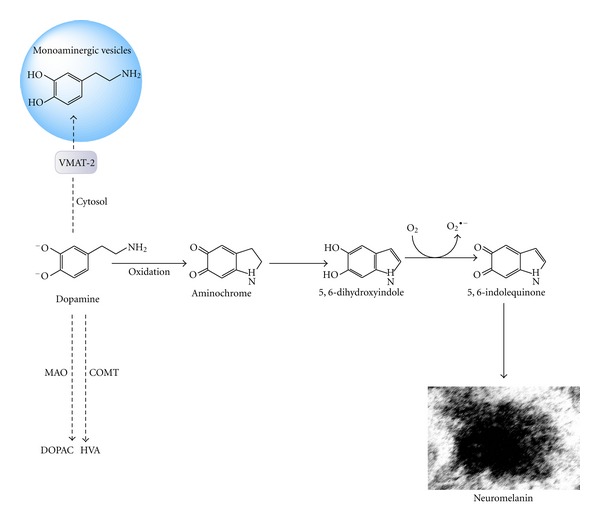
Neuromelanin formation. Dopamine is oxidized to aminochrome, which tautomerizes to 5,6-indolequinone and undergoes polymerization to form the dark pigment neuromelanin.

Aminochrome is the precursor to neuromelanin because neuromelanin formation is dependent on the rearrangement of aminochrome to 5,6-dihydroxyindole, which is then oxidized to 5,6-indolequinone followed by further polymerization to form neuromelanin [25] (Figure 4). Postmortem studies using healthy subjects have shown that neuromelanin formation is a normal process in substantia nigra. Furthermore, this pigment is located in intact dopaminergic neurons because it is formed during the overtime and accumulates with age [26]. Neuromelanin acts as a chelator for metals [27, 28], indicating that this molecule plays a neuroprotective role. Neuromelanin accumulates in double membrane vacuoles, preventing neurotoxic effects of free neuromelanin in cells exposed to this pigment [29, 30].

### 3.2. Formation of Aminochrome and Protein Adducts

 Aminochrome forms adducts with proteins such as *α*-synuclein [31], stabilizing and inducing the formation of neurotoxic protofibrils [32]. In familiar PD, the formation of neurotoxic *α*-synuclein protofibers is dependent on a specific point mutation [33]. However, in sporadic PD, the formation of neurotoxic protofibrils appears to be dependent on the ability of aminochrome to form *α*-synuclein protofibrils. Aminochrome is also able to form adducts with mitochondrial complexes I and III, as well as isocitrate dehydrogenase [34], suggesting that this molecule induces mitochondrial dysfunction and a subsequent collapse in energy. Aminochrome also forms adducts with the protein DJ-1 [34], which has been suggested to be involved in the regulation of mitochondrial dynamics. Overexpression of the DJ-1 mutant associated with PD induces a significant increase in fragmented mitochondria, mitochondrial dysfunction, and increased neuronal vulnerability to oxidative stress [35].

**Figure 5:**
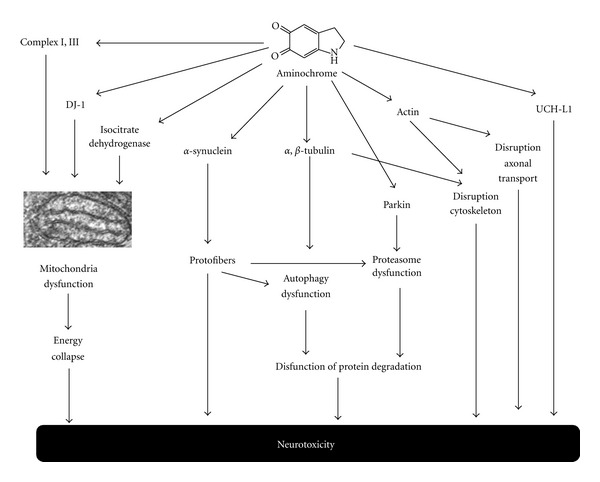
Aminochrome forms adducts with proteins. Aminochrome forms adducts with proteins, such as DJ-1, isocitrate dehydrogenase, and complex I and III of mitochondria, inducing mitochondrial dysfunction and an energy collapse. Adducts formed with *α*-synuclein, induces the formation of neurotoxic *α*-synuclein protofibrils, which inactivate chaperone-mediated autophagy and impair the proteasomal system, resulting in the dysfunction of protein degradation. Aminochrome adducts formed with *α*- and *β*-tubulin induces the aggregation of microtubules that are required for the fusion of autophagy vacuoles with lysosomes. Aminochrome also forms adducts with UCHL-1.

Aminochrome has been reported to disrupt the architecture of the cytoskeleton in cell cultures [15] by forming aggregates with actin and *α*- and *β*-tubulin. Other studies also report the formation of aminochrome adducts with actin and *β*-tubulin [34]. In addition, aminochrome has been shown to form adducts with the ubiquitin carboxy-terminal hydrolase isoenzyme L1 (UCH-L1) [34], which was determined to be associated with familiar PD by a gene mutation (Figure 5).

### 3.3. One-Electron Reduction of Aminochrome

**Figure 6:**
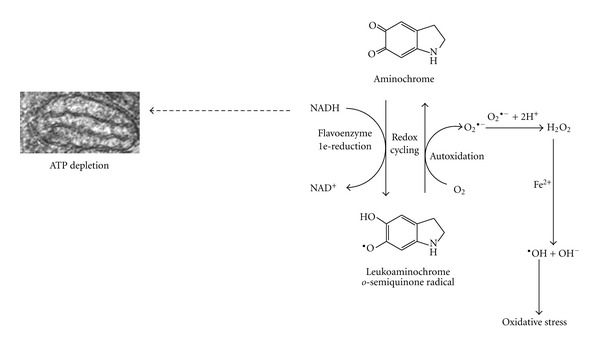
One-electron reduction of aminochrome. The one-electron reduction of aminochrome is catalyzed by flavoenzymes that utilize NADH as an electron donor, generating leukoaminochrome *o*-semiquinone radical. This radical is extremely reactive with oxygen and autoxidizes by reducing molecular oxygen to superoxide radicals. This redox cycling induces oxidative stress and depletes the NADH required for the generation of energy (ATP) in the mitochondria. Redox cycling can also be catalyzed by flavoenzymes that utilize NADPH as an electron donor, depleting the NADPH required for the reduction of oxidized glutathione.

Aminochrome can undergo a one-electron reduction by flavoenzymes that utilize NADH or NADPH as an electron donator to generate a leukoaminochrome-*o*-semiquinone radical. This radical is extremely reactive with oxygen and autoxidizes to aminochrome under aerobic conditions. Molecular oxygen is then reduced to superoxide radicals, generating a redox cycle between the leukoaminochrome *o*-semiquinone radical and aminochrome [10, 36]. The redox cycling between aminochrome and leukoaminochrome *o*-semiquinone radical plays an important role in aminochrome neurotoxicity because it induces an energy collapse when flavoenzymes utilize NADH, which is required for ATP synthesis in the mitochondria [37, 38]. The use of NADPH in redox cycling also affects the cell because NADPH is required to catalyze the reduction of oxidized glutathione by glutathione reductase, which is an important antioxidant. The neurotoxic effects of this redox cycling are enhanced by the dismutation of superoxide radicals to hydrogen peroxide, the precursor of hydroxyl radicals. The one-electron reduction of aminochrome has been reported to be neurotoxic to catecholaminergic cells [15, 21, 22, 38–45] (Figure 6).

### 3.4. Two-Electron Reduction of Aminochrome

**Figure 7:**
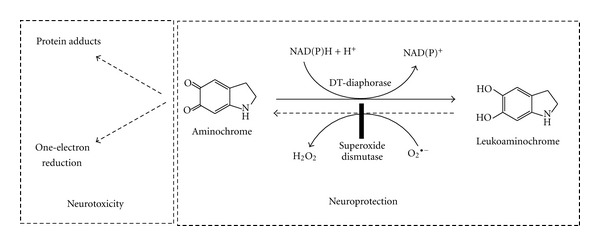
Two-electron reduction of aminochrome catalyzed by DT-diaphorase. DT-diaphorase prevents the participation of aminochrome in neurotoxic reactions, such as the formation of adducts with proteins and one-electron reduction of aminochrome to leukoaminochrome.

Aminochrome can undergo a two-electron reduction by DT-diaphorase (EC.1.6.99.2), a flavoenzyme that uses both NADH and NADPH as electron donors, and the product of this reaction is the hydroquinone leukoaminochrome [11]. Leukoaminochrome can autoxidize in the presence of superoxide radicals. However, the presence of superoxide dismutase in the cytosol prevents leukoaminochrome autoxidation from occurring [36]. We have proposed a protective role for DT-diaphorase against aminochrome neurotoxicity, which is supported by cell culture studies based on the inhibition of DT-diaphorase with dicoumarol and the induced aminochrome neurotoxicity that results from reduced DT-diaphorase expression by siRNA [38, 41, 42, 46]. DT-diaphorase also prevents the formation of *α*-synuclein protofibrils [47, 48] and disruption of the cytoskeleton, which is generally the consequence of forming adducts with actin and *α*- and *β*-tubulin [15]. DT diaphorase immunoreactivity has been observed in dopaminergic neurons and Bergmann glia, astrocytes, and tanycytes [49] (Figure 7).

### 3.5. Aminochrome Conjugation with Glutathione

**Figure 8:**
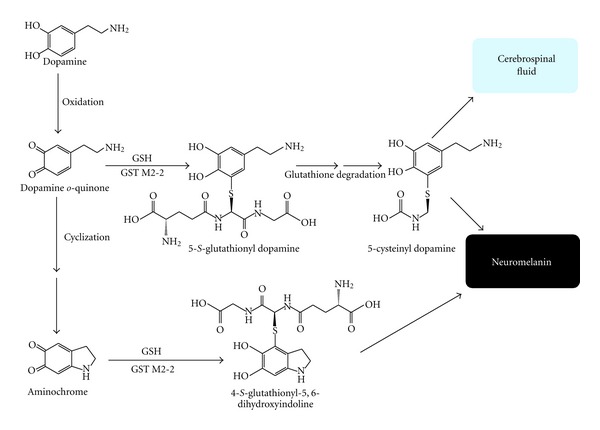
Glutathione conjugation of dopamine *o*-quinone and aminochrome. GST M2-2 catalyzes conjugation of both aminochrome and its precursor dopamine *o*-quinone to 4-*S*-glutathionyl-5,6-dihydroxyindoline and 5-glutathionyl-dopamine, respectively. 5-Glutathionyl-dopamine undergoes degradation to 5-cysteinyl dopamine that have been found in both the cerebrospinal fluid and neuromelanin.

Aminochrome can be conjugated with glutathione by glutathione S-transferase M2-2 (GST M2-2) to 4-*S*-glutathionyl-5,6-dihydroxyindoline. 4-*S*-Glutathionyl-5,6-dihydroxyindoline is stable in the presence of biological oxidizing agents, such as oxygen, superoxide radicals, and hydrogen peroxide [50, 51]. The stability of 4-*S*-glutathionyl-5,6-dihydroxyindoline in the presence of biological oxidizing agents suggest that is a final elimination product. Interestingly, the precursor of aminochrome, dopamine *o*-quinone, is also conjugated by GST M2-2 to 5-glutathionyl-dopamine, preventing the formation of aminochrome [52]. All glutathione conjugates undergo degradation of the tripeptide *γ*-L-Glu-L-Cys-Gly, named the glutathione to cysteil conjugate. Thus, 5-glutathionyl-dopamine is converted to 5-cysteinyl dopamine. Notably, 5-S-cysteinyl-dopamine has been detected in the cerebrospinal fluid of PD patients, dopamine-rich regions of the brain such as the caudate nucleus, putamen, globus pallidus and substantia nigra, and in neuromelanin [53–55]. GST M2-2 also catalyzes the conjugation of dopa *o*-quinone to 5-glutathionyl dopa leads to the degradation of the tripeptide glutathione, generating 5-cysteinyl dopa [56]. Melanoma cells produce and release 5-cysteinyl-dopamine, which is then excreted through the urine [57]. Thus, the conjugation of glutathione must be a protective reaction against aminochrome neurotoxicity (Figure 8).

## 4. PD and Autophagy

Autophagy is an important intracellular bulk degradation and recycling process in which cytoplasmic proteins and organelles accumulate in autophagy vacuoles that are transported into lysosomes [58–60]. Autophagy plays an important role in the elimination of damaged organelles, such as the mitochondria. Autophagy dysfunction has been speculated to play an important role in the pathogenesis of PD [61]. Wild-type *α*-synuclein is degraded by chaperone-mediated autophagy (CMA) and macroautophagy because the inhibition of CMA and macroautophagy lead to accumulation of wild type *α*-synuclein [62]. The expression of the *α*-synuclein mutant A53T induces CMA dysfunction, which is mediated by the expression of *α*-synuclein protofibrils [63]. Interestingly, the pathogenic A53T and A30P *α*-synuclein mutants inhibit their own degradation and that of other substrates [64]. Overexpression of the *α*-synuclein mutant A53T in transgenic animals demonstrates that A53T localizes to mitochondrial membranes as a monomer, inhibiting complex I and increasing mitochondrial autophagy [65]. *α*-synuclein impairs autophagy via Rab1a inhibition, and Rab1a overexpression rescues the autophagy defect caused by *α*-synuclein [66]. A mutation in ubiquitin C-terminal hydrolase L1 (UCH-L1), which is associated with familial PD, was reported to inhibit CMA autophagy by interacting with the lysosomal receptor of CMA LAMP-2A [67]. Parkin has also been shown to promote autophagy of damaged mitochondria by relocalizing into dysfunctional mitochondria with low membrane potentials in mammalian cells [68]. The loss of DJ-1induces a reduction in the mitochondrial membrane potential and an increase in the fragmentation and accumulation of autophagy markers. These effects appear to be mediated by oxidative stress because supplementing DJ-1-deficient cells with glutathione has been shown to reverse these effects on mitochondria and autophagy [69]. Transfection of the common G2019S LRRK2 mutation into SH-SY5Y cells was reported to increase autophagy in both neuritic and somatic compartments [70]. Autophagy activation was observed to restore the mitochondrial membrane potential impaired by rotenone in SH-SY5Y cell lines overexpressing *α*-synuclein [71] and attenuate rotenone-induced toxicity in SH-SY5Y cell lines [72].

## 5. Aminochrome and Autophagy

In Parkinson's disease, autophagy dysfunction plays an important role in the neurodegeneration of dopaminergic neurons containing neuromelanin. Proteins associated with familial PD have been reported to play a role in autophagy dysfunction, such as *α*-synuclein, UCH-L1, and DJ-1 [63, 67, 69]. Mutated *α*-synuclein (A53T) generates protofibrils that inhibit CMA autophagy. As a result, dopamine-modified *α*-synuclein is poorly degraded by CMA and also inhibits the degradation of other substrates using this pathway [73]. Aminochrome was reported to form adducts with *α*-synuclein by binding to the 125YEMPS129 motif of *α*-synuclein and inducing and stabilizing the formation of protofibrils [31]. These observations suggest that aminochrome is involved in the alpha synuclein-dependent inhibition of CMA autophagy because aminochrome induces the formation of *α*-synuclein protofibrils, such as the A53T mutant (Figure 10). Mutated UCH-L1 also inhibits CMA autophagy by interacting with the lysosomal receptor for CMA LAMP-2A [67]. Aminochrome has been shown to also form adducts with UCH-L1 [34]. Little is known about the effects of the aminochrome-induced modification of UCH-L1. However, we speculate that the aminochrome-induced modification of UCH-L1 also impairs CMA autophagy. In addition, aminochrome forms adducts with the protein DJ-1, and the loss of DJ-1 indirectly alters autophagy by interfering with the regulation of oxidative stress [69].

**Figure 9:**
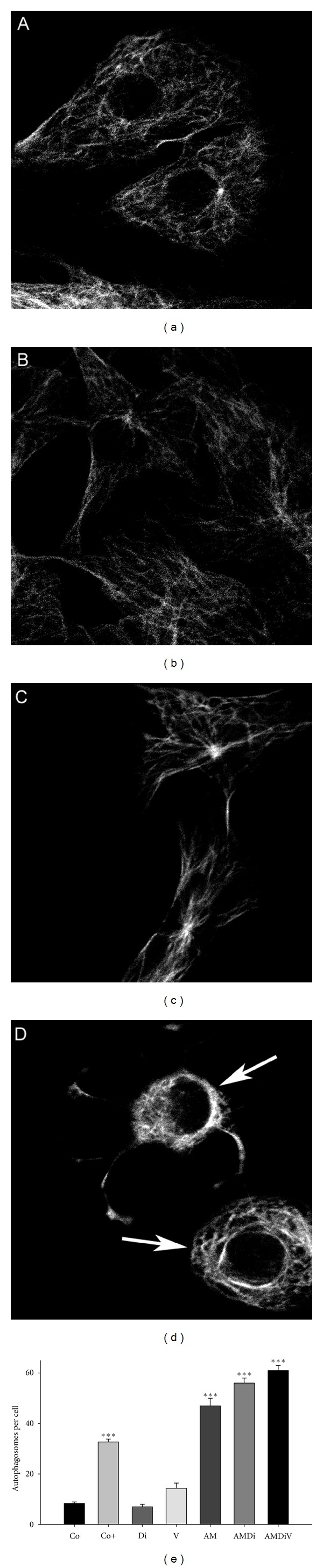
The effects of aminochrome on *β*-tubulin (a–d) and the accumulation of autophagosomes in the cytosol of cells treated with aminochrome (e). RCSN-3 cells were treated with cell culture medium (a), 100 *μ*M dicoumarol (b), 50 *μ*M aminochrome (c), and 50 *μ*M aminochrome and 100 *μ*M dicoumarol (d) and incubated for 48 h as described by Paris et al. 2010 [15]. In (e) the number of autophagosomes per cell was determined by incubating RCSN3 cells for 24 h with cell culture medium (Co), 100 *μ*M dicoumarol (Di), 10 *μ*M vinblastine (V), 20 *μ*M aminochrome (AM), 20 *μ*M aminochrome and 100 *μ*M dicoumarol (AMDi), 20 *μ*M aminochrome and 100 *μ*M dicoumarol and 10 *μ*M vinblastine (AMDiV). As a positive control, cells were incubated with cell culture medium without bovine serum (Co+). The statistical significance was assessed using ANOVA for multiple comparisons and Student's *t* test (***P < 0.001). This experiment was performed as described by Paris et al. 2011 [40].

**Figure 10:**
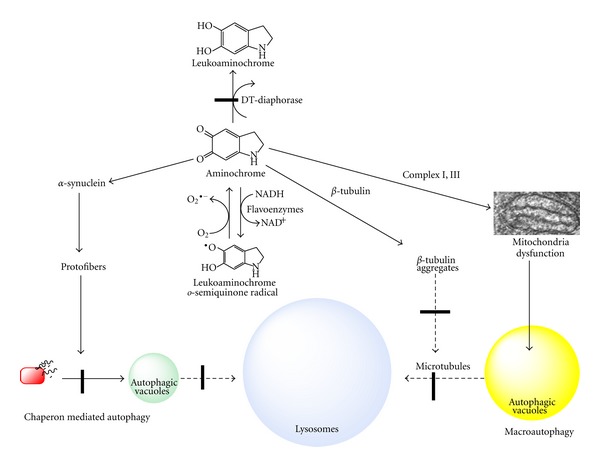
Possible role of aminochrome in autophagy. Aminochrome induces the dysfunction of autophagy by forming adducts with the following: (i) alpha synuclein, which inactivates chaperone-mediated autophagy; and (ii) *α*- and *β*-tubulin, inducing the aggregation of microtubules, which are essential for the fusion of autophagy vacuoles with lysosomes.

Microtubules are an important component of the cytoskeleton, which are composed by subunits of *α*- and *β*-tubulin and normally exist as dimers. Microtubules also play a role in the formation of autophagosomes and fusion of autophagosomes with lysosomes [74, 75]. Aminochrome forms adducts with *β*-tubulin [34] and the aminochrome one-electron reduction product when DT-diaphorase is inhibited by dicoumarol. This inhibition leads to the disruption of the cytoskeleton by disrupting the **α**- and **β**-tubulin network and its aggregation around the cell membrane ([15]; Figure 9). Thus, we speculate that aminochrome prevents the fusion of autophagosomes with lysosomes by inducing the aggregation of microtubules. We hypothesize that the number of autophagosomes will increase in the cytosol when aminochrome inhibits the fusion between autophagosomes and lysosomes by preventing the formation of normal microtubules because of the formation of aminochrome adducts with *α*- or *β*-tubulin (Figure 10). Incubation of RCSN-3 cells with 20 *μ*M aminochrome in either the presence or absence of 100 *μ*M dicoumarol induced a significant increase in the number of autophagosomes in the cytosol (6- and 9-fold, resp.; Figure 9). These results support aminochrome playing a role in autophagy dysfunction. Aminochrome forms adducts with *α*- or *β*-tubulin, preventing the fusion of autophagosomes and lysosomes that lead to an increase in the number of autophagosomes in the cytosol [15]. Furthermore, these data support the observation that aminochrome induces a significant increase of GFP-LC3 positive staining in cells treated with aminochrome [41]. Aminochrome has also been reported to inactivate parkin, an ubiquitin ligase of the proteasomal system, by forming adducts with parkin [14] as well as inhibit the proteasome [76]. All of these results support the involvement of aminochrome in the dysfunction of protein degradation.
